# Enhanced Engraftment of a Very Low-Dose Cord Blood Unit in an Adult Haemopoietic Transplant by Addition of Six Mismatched Viable Cord Units

**DOI:** 10.4061/2010/431909

**Published:** 2009-08-26

**Authors:** Stephen J. Proctor, Catherine E. Chapman, Rachel Sharples, Helen L. Lucraft, Jennifer Wilkinson, Jane Conn, Peter G. Middleton

**Affiliations:** ^1^Haematological oncology Group, Academic Haematology, Medical School, Newcastle University, Newcastle upon Tyne NE2 4HH, UK; ^2^Medical Department, Newcastle Blood Centre, NHS Blood and Transplant, Holland Drive, Newcastle upon Tyne NE2 4NQ, UK; ^3^Newcastle NHS Hospital Foundation Trust, Freeman Road, Newcastle upon Tyne NE7 7DN, UK; ^4^Northern Centre for Cancer Treatment, Newcastle NHS Hospital Foundation Trust, Freeman Road, Newcastle upon Tyne, NE7 7DN, UK

## Abstract

The report describes the feasibility of the addition of multiple viable HLA-mismatched unrelated cord blood units, to a low cell number matched unrelated cord, to assist clinical engraftment. An ablative stem cell transplant was performed in an adult with relapsed acute lymphoblastic leukaemia (ALL), using a single HLA-matched cord blood unit (mononuclear cell dose 0.8 × 10^7^), supported by six mismatched cord blood units (one unit per 10 kg recipient weight). No adverse reaction occurred following the infusion of mismatched units and engraftment of the suboptimal-dose matched unit occurred rapidly, with no molecular evidence of engraftment of mismatched cords. Early molecular remission of ALL was demonstrated using a novel PCR for a mitochondrial DNA mutation in the leukaemic clone. The cell dose of the matched cord was well below that recommended to engraft a 70 kg recipient. We suggest that a factor or factors in the mismatched cords enhanced/supported engraftment of the matched cord.

## 1. Introduction

The value of cord blood (CB) as a source of stem cells for transplantation in adults has been enhanced by the key observations that engraftment and outcome can be facilitated by the use of two partially matched cords [1, 2]. This approach has been used to increase overall stem cell number and appears to create a pattern of engraftment, and clinical outcome similar to that of matched unrelated bone marrow transplant of adult to adult [3]. CD 34+ cell dose has been considered critical for safe engraftment and the threshold level recommended is >1.7 × 10^5^ kg [4], or a total doses of nucleated cell (TNC) >3.7 × 10^7^ have been considered necessary [5], hence the use of two cords, to overcome this dose problem when transplanting adults. However, it is usual that only one of the infused cords engrafts which raises the question of additional factors in the nonengrafting cord that might facilitate the engraftment process. 

 Evidence has now emerged in a NOD/SCID mouse model, that use of two cords will enhance engraftment even when compared with single units containing the same cell numbers as the combined cords [6]. The authors of these studies conclude that the results indicate that engraftment may be enhanced by addition of a second unrelated CB and may be attributed to cell dose increase or due to as yet unknown factors providing a graft-facilitating effect, since engraftment often occurs in this setting with only one of the pair of cords successfully engrafting. 

 Additionally, observations of experimental transplants in humans using multiple totally mismatched cords, which demonstrate some engraftment [7, 8], also provide intriguing evidence to suggest that novel approaches to enhance cord engraftment are warranted, in otherwise fatal clinical situations. 

 In this preliminary report we describe the use of a very low dose, matched cord unit (0.8 × 10^7^/kg mononuclear cord cells), utilised successfully to engraft an adult patient supported by the use of multiple (six) mismatched viable cords that did not themselves engraft.

## 2. Case History

In 2001 a 31-year-old white male (wt 70 kg) presented with acute lymphoblastic leukaemia in November 2000. Peripheral WBC was 82 × 10^9^/L. The tumour was CD10+, CD13+, CD19+, CD20+, CD33+, CD34+, HLADR+, and TDT+. Standard cytogenetics failed, but cells were BCR/ABL negative by PCR. He was treated with an aggressive multiagent chemotherapy schedule (Northern Region Haematology Group NEALLVI) for a six-month period. An attempt at the end of therapy to harvest peripheral blood stem cells failed. There was no sibling donor or adult matched unrelated haemopoetic stem cell (HSC) donor available. Six months after completing the protocol he relapsed. Reinduction with further aggressive chemotherapy (FLAG-fludarabine, cytosine arabinoside and GCSF) was completed causing sustained and irrecoverable marrow hypoplasia with persistence of small numbers of leukaemia blasts. 

 With no adult HSC donor available we identified a fully HLA-matched male cord blood unit (total nucleated cell dose 1.4 × 10^7^/kg, mononuclear cell dose 0.8 × 10^7^/kg), CD 34 dose unavailable. This matched unit was blood group O, and the recipient was blood group A. This single unit was (in 2002 the date of transplant) considered insufficient to guarantee engraftment in a 70 kg recipient. After detailed discussions with patient and relatives, and considering preliminary data from Gryn et al. [7], six additional totally HLA-mismatched, blood group matched, female cord blood units were selected from the Newcastle Cord Bank to increase the stem cell component. 

 Preconditioning consisted of Rituximab (anti-CD20 antibody) for four doses (325 mg/sq·m per dose), over 8 days. Marrow blast content was unchanged directly after this treatment. Additional preconditioning consisted of intravenous Melphalan 120 mg/m^2^ plus TBI of 1200 cGy in six fractions over three days. Intrathecal methotrexate was also utilised. The HLA-matched cord unit was given prior to the nonmatched cord units to facilitate predominant engraftment with the matched element. The remaining cord units were all given over a one-hour period four days later. Nucleated cell content of these units is shown in Table 1.

### 2.1. Engraftment

Details of the single matched cord engraftment are tabulated in Table 2. White blood cell reconstitution showed a level of 1.3 × 10^9^/L on day +28 and a staggering granulocyte count of 7.45 × 10^9^/L on day +32 which reduced subsequently as GM CSF (started on day +7) was discontinued. All white cells were from the matched cord unit. Platelet engraftment, as anticipated, was slow and did not reach a level of 30 × 10^9^/L unsupported until day 120 (Table 2). 

Molecular markers of the leukaemic clone (immunoglobulin heavy chain markers (IgH/VH1/VH2) and T cell gamma gene rearrangement (courtesy of Dr. L. Foroni, Royal Free Hospital, London) disappeared in clinical remission after chemotherapy, returning on subsequent relapse. These markers were no longer identifiable in the day 100 sample, post cord blood transplant. 

 Genotype tracking of all transfused mismatched cord blood units used was performed by VNTR analysis using three microsatellite loci (TNFa and TNFd (6p21.3) and the CTLA4 locus (2q33)). All transfused units had unique genotypes using these markers (Table 1). On Day +5 trace of the patient's genotype was detectable and subsequently became undetectable. The HLA-matched cord unit genotype was detected from day +7 with only weak signals from any of the nonmatched cord genotype between day's 5–7 after which they disappeared. (sensitivity of detection of this analysis is 2%–5 %). 

 In a separate study of mitochondrial mutations in haematological malignancy, the leukaemic cells from this patient were shown to have a somatic mitochondrial DNA mutation in the *cytochrome b* gene (He et al. [9]). A sensitive specific PCR assay for detection of the mutation showed the mutation to be present at diagnosis and in relapse cells. After cord unit transplant there was no evidence of this mutation in the bone marrow, either at day +28, +100, or +140 indicating molecular remission at that stage (sensitivity of detection <0.1% using a quantitative SYBR Green real-time PCR assay, see He et al., [9] for details). The day 100 bone marrow was populated only by the matched cord (FISH 100% chimaerism).

## 3. Discussion

Cord blood transplant can be safely considered in adults using a single suitable cord unit, and results are sufficiently good to recommend the approach when there is no suitable adult donor [10]. The issue relates to the cell dose and speed and safety of engraftment, and anything that can improve this consistently has clear interest. Recent results have confirmed that it is a major problem to transplant with <2 × 10^7^/Kg nucleated cells [11, 12] though Takahashi et al. did see delayed engraftment in seven transplants with 1–2 × 10^7^/Kg. 

 Clearly, there appears to be much merit in double cord transplant with partial matching of the units [1–3], and the striking observation of Gryn et al. [7] confirmed in a later report by Lister et al. [8] that sustained engraftment can occur with completely mismatched cord units, led to the approach described here, that of mismatched cord units supporting a small matched cord unit. Since this procedure occurred in 2002 before double cord transplants with partial matching were established, we were uncertain as to the number of additional completely mismatched cords that would be required. The choice of six additional cords related to the small amount of data on using completely mismatched units alone (7), so this number was adopted arbitrarily to ensure that a high total dose was delivered. The choice of cord HLA type between additional cords was not a consideration; the main features were they were the same blood group as the recipient; all female donors aid tracking and CMV negative. 

 Clinically this procedure was an uncomplicated stem cell transplant with no immediate or delayed reaction to the mismatched cord units. Immunosuppression was with cyclosporine A alone, which was discontinued on day +30, and no acute graft versus host disease occurred. 

 There was rapid engraftment of the granulocyte series (Table 2) and only transitory evidence of the DNA from the other six cord units early in the posttransplant period. In double cord transplants the pattern of engraftment observed is that usually there is the emergence of an individual single cord as the dominant element, but there can be mixed chimeras. In double cord transplants, however, there is usually at least partial matching with the patient and the other cord [1]. 

 The speed and extent of myeloid engraftment of the low-dose HLA-matched CB unit in this patient, with neutrophils of 7.45 × 10^9^/l on day 32, raises the question of whether the other cord units supported engraftment as suggested in the SCID model [6]. The use of seven cords in total is related to the observation of Gryn et al. [6] of using one cord unit per 10 Kg body weight of the recipient. Applying this approach in our patient was uncomplicated and provides a model for further experimental applications. The subsequent course was punctuated by an encephalopathic illness caused by HHV6 infection, which was satisfactorily treated using Foscarnet over several weeks. 

 The novel mitochondrial DNA mutation which was present in the leukaemic clone enabled us to assess the state of biological remission in this patient at an early stage of posttransplant. Haematological remission in advanced ALL rarely occurs after chemo-radiotherapy and matched allogeneic transplant in relapse. The achievement of “molecular remission” in this case, at an early stage posttransplant, suggests that if GVHD/GVL had occurred, then the remission might have been enhanced. Unfortunately, after 8 months in remission, the leukaemia relapsed, with return of all the clonal molecular markers. (Patients transplanted in relapse have the highest likelihood of disease relapse following transplant, irrespective of the source of stem cells or the modality of treatment [12]). 

 Clearly any approach which can lead to quicker and safer engraftment of cord stem cells in adults where the matched units are very small is worth further exploration and suggests that the double cord approach success may not just be related to increase of stem cell numbers. It seems possible that our findings in this case might tempt the experimentalists with an animal model to further define the possible biological mechanisms operating in multicord transplant.

## Figures and Tables

**Table 1:** Details of transplanted cord units. Cellular characteristics and HlA types of the cord units used in the transplant are given. No attempt was made to have matching tissue types in the unmatched cords. The matched cord differed from the patient in blood group Patient A+ matched cord O+. Tissue type of matched cord was identical at Class 1 DR, and DP were fully matched but there was a DQ mismatch. All cord donations were female and CMV antibody negative Genotypes are the allele ID's for the markers used at the CTLA4 and TNFA loci (refs). The matched cord cell dose was substantially less than the recommended dose for engrafting an adult (0.8 × 10^7^/kg) but engraftment of granulocytes was swift and uncomplicated. NK-not known.

Sample description	Blood group and Hla types of transfused cord units	Total NUC cell count × 10^7^	Nucleated cell count 10^7^/kg	MNC × 10^7^ Total	CD34 ×10^8^ Total	CTLA4 microsatellite genotype	TNF microsatellite genotype
Patient leukaemia	—	—	—	—	—	27, 28	a2,7; d3,4

Matched cord	O Pos and A29, A68, B44, B1402/04/06 Cw08, Cw1601, DRB1, DQB1, DPB1	102	1.46	56.2 (0.8 ×10^7^/kg)	- NK	16, 19	a2,7; d4,4

Nonmatch cord 1	A Pos and A2, A11, B8, B35, Cw7, Cw4/18, DR1	66.6	0.95	- NK	0.36	8, 16	a6,10; d3,3

Nonmatch cord 2	A Pos and A1, A11, B18, B38, Cw12, Cw7, DR13, DR15	132.8	1.89	- NK	0.292	8, 8	a2,2; d1,4

Nonmatch cord 3	A Pos and A1, A26, B8, B49, Cw7, DR1, DR17	159.8	2.28	- NK	0.639	8, 22	a2,5; d1,3

Nonmatch cord 4	A Pos and A1, A2, B8, B27, Cw2, Cw7, DR17	80.2	1.15	- NK	0.217	8, 19	a2,2; d1,4

Nonmatch cord 5	A Pos and A2, B44, B60, Cw5, Cw10, DR13, DR15	64.6	0.92	- NK	0.058	19, 22	a4,11; d3,3

Nonmatch cord 6	A Pos and A2, A3, B7, B60, Cw7, Cw10, DR4, DR17	91.4	1.31	- NK	0.165	8, 8	a2,11; d3,4

Patient blood +21 days		—	—	—	—	16, 19	a2,7; d4,4

**Table 2:** Pattern of haematological engraftment. Cell counts and tracking data for the post transplant period. A relative monocytosis and eosinophillia has persisted throughout the engraftment period. Marrow at day 28, 100 and 140 demonstrates molecular remission using MRD markers.

(×10^9^/l)	Day 1	Day 15	Day 21	Day 28	Day 32	Day 36	Day 40	Day 50	Day 75	Day 100	Day 140
Total WCC	0.00	0.4	0.6	2.3	8.8	6.8	5.0	10.3	5.6	4.6	11.1
Neutrophils	0.03	0.04	0.33	1.3	7.45	3.5	2.46	7.37	2.53	1.98	5.97
Lymphocytes	0.01	0.29	0.15	0.26	0.19	0.84	0.8	0.29	0	1.0	1.03
Monocytes	0.00	0.02	0.16	0.65	1.02	1.43	1.14	1.63	0.91	0.95	2.60
Eosinophils	0.00	0.00	0.00	0.12	0.02	1.1	0.56	0.8	1.78	0.3	1.4
Basophils	0.00	0.00	0.00	0.01	0.15	0.01	0.08	0.14	0.14	0.13	0.05
Haemoglobin	8.7	11.1	10.8	10.4			11.1	10.6	10.4	10.0	11.6 (unsupptd)
Platelets	22	24	18	42			14	8	14	36	58 (unsupptd)
Blood gp A-mitochondrial DNA mutation (marrow) +ve IgH (V2) +ve				Mitochondrial DNA mutation (marrow) −ve				Blood Group O + ve confirmed		Mitochondrial DNA mutation (marrow) −ve IgH (V2) −ve	Mitochondrial DNA mutation (marrow) −ve
